# Research on the Induced Electrostatic Discharge of Spacecraft Typical Dielectric Materials under the ESD Pulse Irradiation

**DOI:** 10.3390/ma15062115

**Published:** 2022-03-13

**Authors:** Xiaofeng Hu, Jianping Zhang, Ming Wei, Yingying Wang

**Affiliations:** 1 National Key Laboratory of Electromagnetic Environmental Effects, Army Engineering University, Shijiazhuang 050003, China; snowfox2270@163.com (X.H.); 13784397599@139.com (M.W.); wangying1886103@163.com (Y.W.); 2 Unit 32140 of PLA, Shijiazhuang 050061, China

**Keywords:** electrostatic discharge, dielectric materials, electric field pulse radiation, discharge frequency

## Abstract

Based on polyimide (PI), epoxy resin (EP), polytetrafluoroethylene (PTFE) typical dielectric materials used in spacecraft, a research platform for charge-discharge experiment under strong field was established. The influence of irradiation field strength, beam energy, dielectric thickness, etc., on electrostatic discharge characters of dielectric materials were studied and summarized. The results show that with the increase of field strength, the frequency of induced electrostatic discharge increases, the accumulated charge on the dielectric surface decreases, and the dielectric surface potential decreases; Under the same conditions, PI has the lowest discharge frequency and the highest surface dynamic balance potential. The thicker the dielectric material, the lower the discharge frequency, the higher the surface dynamic equilibrium potential; The shape and size of the background electrodes also affect the discharge frequency. When the area is the same, the sharper the electrode edge, the higher the discharge frequency. When the shape is the same, the larger the grounding area, the higher the discharge frequency. By proposing the induced discharge test method, the function mechanism of spatial environmental factors on the electrostatic discharge of typical dielectric materials are obtained. Comparative analysis on electrostatic properties of different dielectric materials can provide data reference and technical support for spacecraft electrostatic safety and protection.

## 1. Introduction

Electrostatic discharge (ESD) of spacecraft in electromagnetic environment is a very complex process, which is related to space environment, spacecraft structure, size and material properties [1,2,3]. Due to the existence of space radiation environment, spacecraft will be affected by natural hazards such as electrostatic discharge and micro discharge caused by secondary electron multiplication. The instantaneous current generated by electrostatic discharge can cause oxidation, carbonization or breakdown of spacecraft surface materials, resulting in serious interference or even failure of communication and navigation systems [4,5,6,7]. For space equipment, the electrostatic discharge induced by strong electromagnetic field often occurs in the low charging potential area of spacecraft surface materials, cables or some special structural components [8,9,10]. In general, the electric field in the sensitive area of low charge potential on the spacecraft surface is low, the electron energy is lower than the ionization energy of molecules or atoms, and the collision ionization probability is low [11,12,13,14]. When subjected to strong electromagnetic field, low pressure gas is excited to produce dense plasma, thus reducing the discharge threshold and inducing electrostatic charge discharge [15,16,17].

Due to the use of a large number of dielectric materials in a real spacecraft, its influence on the induced discharge of strong electromagnetic field cannot be ignored. Under high vacuum conditions, the dielectric surface of spacecraft is often charged to tens of thousands of volts by high energy charged particles. When the potential difference between the surface material and the grounding structure of spacecraft reaches a certain degree due to the accumulation of charge, it may cause pulse discharge and charge-discharge effect on the spacecraft surface, which seriously affects the safe operation of the spacecraft [18]. Therefore, it is necessary to study the discharge mechanism of dielectric materials induced by strong electromagnetic field. At present, researchers have conducted a large number of ground simulation studies on the charged behaviour of spacecraft dielectric materials, and have obtained a series of research results [19,20,21]. Q.S. Su et al. studied the influence of area and thickness of polyimide (PI), epoxy resin (EP) on their charging rate and equilibrium potential, and obtained that the equilibrium potential increased with the increase of the thickness and area of material [22]. D.T. Li studied the influence of shape, thickness and ground structure of dielectric materials on charging characteristics [23]. R.H. Quan et al. studied the variation of deep charging equilibrium potential and equilibrium time with electron beam intensity and dielectric resistivity of PTFE and other dielectric materials [24]. However, there are few studies on the charge-discharge law of dielectric materials induced by strong electromagnetic field, and the discharge characteristics of different dielectric materials are also lack of analysis and comparison.

In this paper, PI, EP, polytetrafluoroethylene (PTFE) commonly used in spacecraft are used as test objects and are irradiated and charged in a vacuum environment using high energy electron beam to simulate the space charged environment. ESD field pulse is used to simulate the electric field irradiation of the space environment without considering the effect of magnetic field and plasma concentration, which will be analysed in further research. The aim of this work is to: (1) explore the basic rules of charge-discharge characteristics of dielectric materials under different spatial environmental factors, such as high energy electron beam irradiation and ESD pulse radiation; (2) obtain the influence law of the type and structure of dielectric materials on electrostatic properties; (3) provide the data reference and technical support for electrostatic safety and protection of space vehicles.

## 2. Materials and Methods

### 2.1. Setting of Dielectric Materials

**Figure 1:**
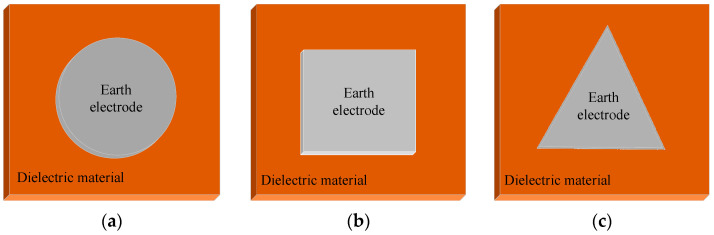
Setting of ground electrode for dielectric materials, (**a**) Circular electrode, (**b**) Square electrode, (**c**) Triangular electrode.

PI, EP and PTFE materials are set to different thicknesses (0.1 mm, 0.2 mm and 0.5 mm) for test. The aluminium platinum (Al-Pt) metal electrodes (Chuangsheng, Shijiazhuang, China) grounded on the back of the dielectric material are round, square and triangular as shown in Figure 1, whose sizes are set to a specific value. The discharge law of dielectric materials with different types, thicknesses, shapes and sizes of ground metal coatings can be studied.

### 2.2. Experimental Apparatus

**Figure 2:**
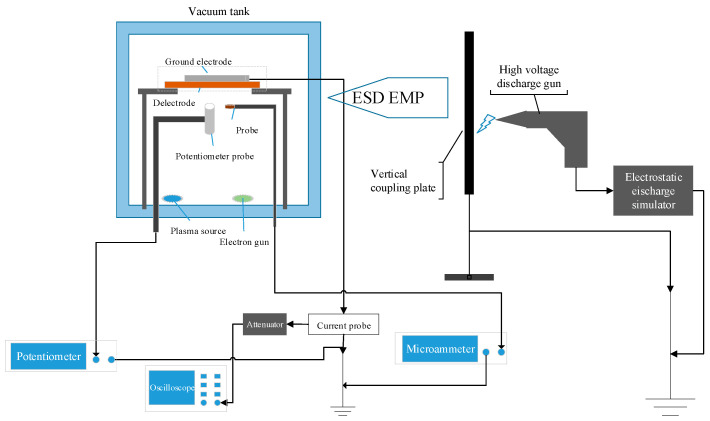
Connection diagram of ESD induced by ESD field pulse on dielectric materials.

**Figure 3:**
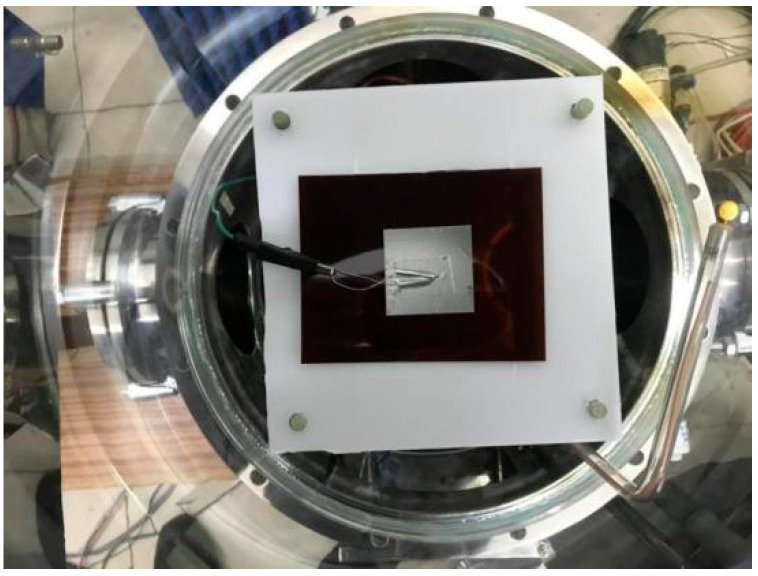
ESD test of dielectric materials induced by ESD field pulse.

The experiment uses the induced electrostatic discharge test system designed by our group to provide conditions for high vacuum and high energy electron beam irradiation and uses the ESD pulse as the radiation field of inducing electrostatic discharge to simulate the strong electrostatic field in space. The experimental principle is shown in Figure 2. Keep the vacuum tank and the vertical coupling plate in the same position as that calibrated in the radiation field strength, and the test site was shown in Figure 3.

Based on the test schematic diagram, the specific operation is orderly to place the sample, connect the equipment, pump the vacuum tank under the vacuum state of 10^−^^3^ Pa, adjust the electron gun to the high voltage source, and then set the energy density of the electron beam to a fixed value. The range of electron beam energy is 15–30 keV, and beam density is 5–9 nA/cm^2^. During the test, the sample was irradiated and charged by the electron beam for a total of 300 s, the surface potential of which was observed and recorded every 10 s with a EST102 vibrating capacitive electrostatic meter. At the same time, the CT-1 current probe (with a bandwidth of 25 kHz–1 GHz and a volt-ampere output characteristic of 5 mV/1 mA) was used to collect the current signal on the ground circuit of the electrodes discharge. The attenuator (60 dB) was used to access the input port of the oscilloscope (Tektronix TDS7404B, Shanghai, China, with a bandwidth of 4 GHz and a sampling rate of 20 GHz) to observe and record the induced discharge waveform and the discharge frequency (discharge times per unit minute). In addition, Agilent 34460A ammeter, Shanghai, China connected probe was used to monitor the current intensity irradiated by the electron beam from electron gun.

The sample was irradiated by ESD pulse generated by the discharge between the electrostatic discharge simulator and the coupling plate, once per second for a total of 300 s. Using the above method to record the induced discharge under different ESD field intensity conditions. In order to exclude the interference of the cumulative effect of dielectric discharge, the experiment was repeated 5 times with or without contrast condition, and the average value was taken for analysis.

### 2.3. Calibration of Radiation Field Intensity

**Figure 4:**
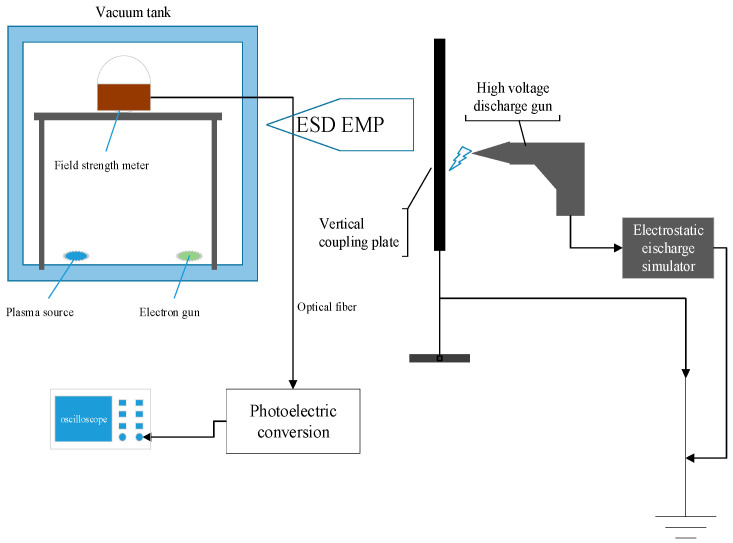
Pulse electric field test system.

**Figure 5:**
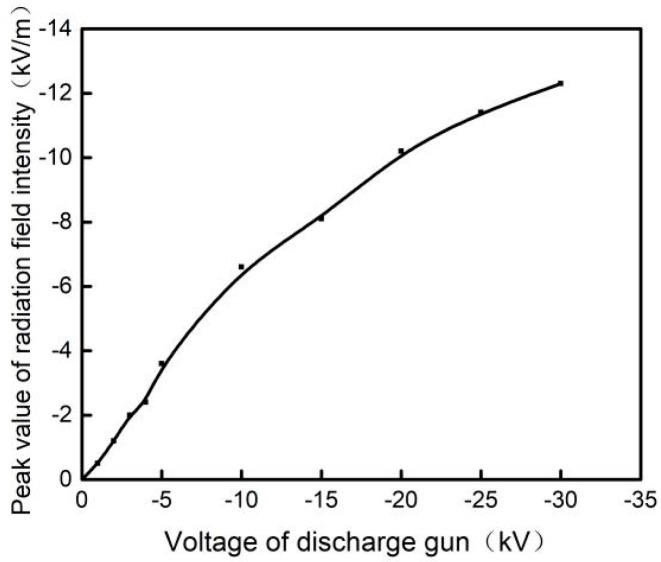
Variation trend of peak value of ESD field with discharge gun voltages.

Figure 4 is the connection diagram of ESD radiation field strength test. Firstly, the relative position of the vacuum tank and the vertical coupling plate was fixed according to the actual situation of the test, and the broadband ESD radiation field test system (3.5 Hz-GHz) was placed at the position of the electrode during the test, and then the ESD Simulator voltage was set at −5 kV, −10 kV, −15 kV, −20 kV, −25 kV and −30 kV. The broadband ESD radiation field test system and oscilloscope were used to test the radiation intensity of ESD field produced by discharge gun. The communication between the field strength meter and the oscilloscope was carried out through optical fibre to prevent electromagnetic pulse from interfering with the oscilloscope and affecting the test accuracy. The peak field strength of ESD field under different discharge gun voltages is listed in Table 1, and the corresponding change trend is shown in Figure 5.

It can be seen from the above figure that the radiation field strength of ESD EMP increases gradually with the increase of the discharge gun voltage, and the radiation field strength is close to proportional to the discharge gun voltage.

## 3. Results

### 3.1. Analysis of Typical Induced Discharge Current Waveform under Different Electric Field Irradiation

**Figure 6:**
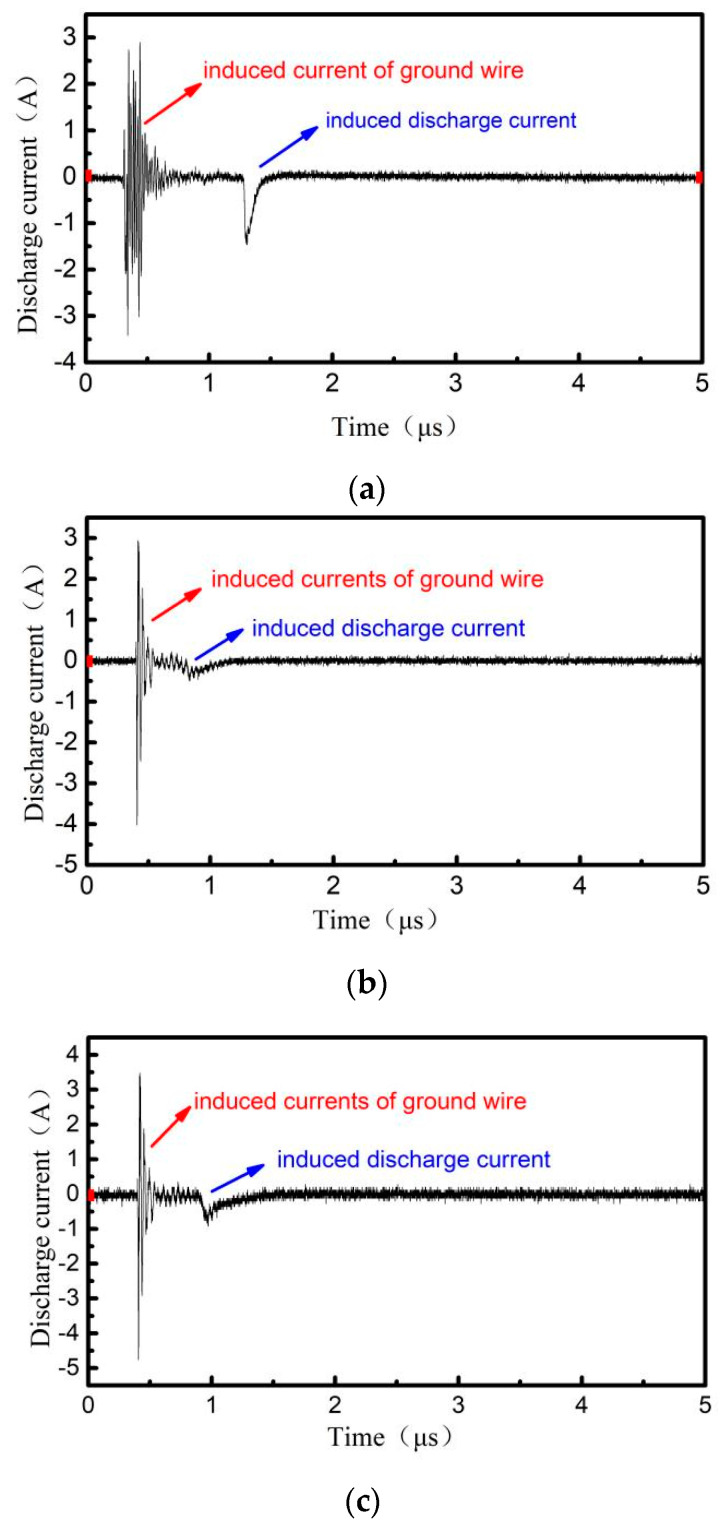
Typical induced discharge waveforms of 3 kinds of dielectric materials under ESD radiation, (**a**) PTFE, (**b**) PI, (**c**) EP.

Under the radiation field with a peak field strength of 12.3 kV/m in ESD field pulse, the typical induced discharge waveforms of PTFE, PI and EP with 0.5 mm thickness are shown in Figure 6, which in conditions that the charge electron beam energy is 30 keV and the beam density is 17 nA/cm^2^ using the equipment in the Figure 2.

From Figure 6, it can be seen that the discharge waveform consists of two parts: a high frequency pulse waveform in the left part and a low frequency pulse waveform in the right one. The left part is very similar to the coupling signal of the electrostatic discharge radiation field on the line circuit, and the pulse amplitude and characteristics are the same. It can be determined that the left part is a ground line induction signal, which is the basis of induced discharge. The waveform on the right is the induced electrostatic discharge waveform.

The mechanism of induced electrostatic discharge is as follows. For PI, PTFE and EP as typical polymer insulating materials commonly used in spacecraft, thermal ionization occurs inside them, and positive and negative charges are separated to generate carriers. However, the generated carriers have much less heat than the band gap of the barrier trap, most of which cannot generate barrier penetration effect, so the conductivity of the polymer is very small [16]. When irradiated with an external strong field, the ability of the internal carriers to move directionally is enhanced, and the probability of internal carriers successfully crossing the barrier trap is improved, that is, the tunnelling effect [25]. Therefore, the conductivity of PI, PTFE, EP and other dielectric materials will increase under the action of strong electric field.

### 3.2. Study on Surface Charging Potential Characteristics of Spacecraft Dielectric Materials under Strong Field

#### 3.2.1. Effect of Radiation Field Strength on Surface Charging Potential of Dielectric Material

**Figure 7:**
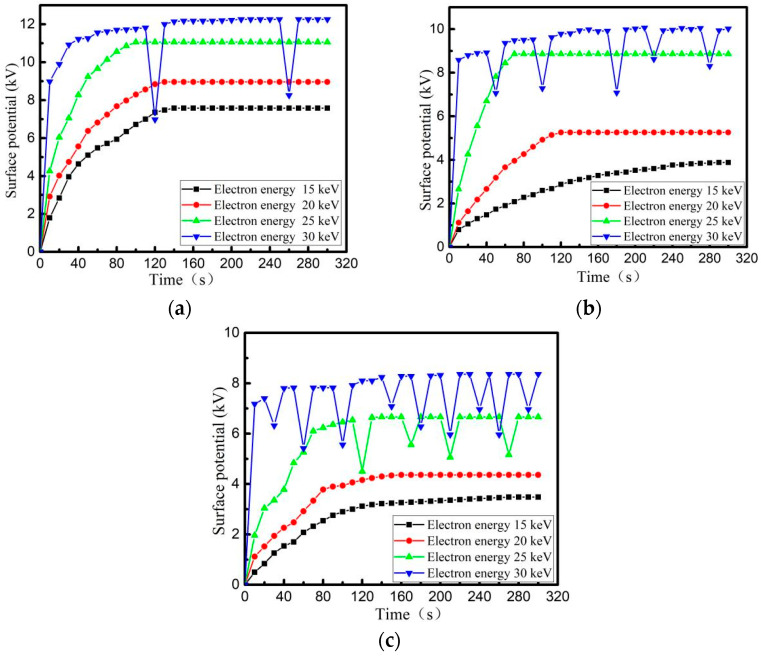
The change law of charging potential on dielectric surface without external field radiation (**a**) PTFE, (**b**) PI, (**c**) EP.

**Figure 8:**
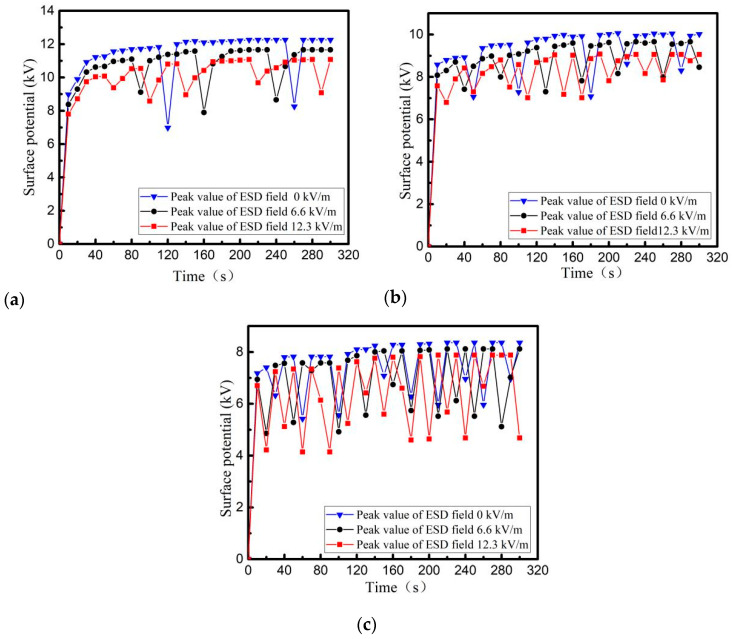
Changes of charging potential on dielectric surface under different radiation field strength (**a**) PTFE, (**b**) PI, (**c**) EP.

When the beam density of the electron beam is 5 nA/cm^2^, PI, PTFE and EP with a thickness of 0.2 mm and back-grounded Al-Pt of 5 × 5 cm^2^ are irradiated and charged in the surface area of 10 × 10 cm^2^. The surface potential is measured shown in Figure 7. After the material surface is decomposed by plasma, the specimens are charged by electron beam with energy of 30 keV, and then radiated once interval of 1 s by an ESD field with different intensities. The variation trend of surface potential of the three media within 300 s is shown in Figure 8.

From Figure 7, it can be seen that the surface potential of PI, PTFE and EP materials increases gradually when charged by irradiation with high-energy electron beams. Although the surface potential of three ones decreases due to ESD leading to charge discharge, the surface potential of the media still increases gradually, which finally reaches a dynamic balance limit (surface charge balance potential). The surface balance potential also increases with increasing energy of the irradiated electron beam. From the comparison in Figure 7 and Figure 8, it can be seen that when ESD irradiation is applied to a material with saturated surface charge, the discharge frequency of material increases, that is, the induced electrostatic discharge is generated on the surface of the dielectric material under the action of the radiation field. At the same time, with the increase of radiation field strength, the number of evoked electrostatic discharges increases, the accumulated charge on the dielectric surface decreases, and the dielectric surface potential drops.

Analysis: This phenomenon can be explained by the following two aspects: on the one hand, according to energy band theory, the polymers’ forbidden band is wide and conduction band is narrow. With the increase of irradiation time, the incident electron can form a large number of localized states and the transport charge inevitably falls into the shallow and deep traps in the forbidden band. The deposition charge in the trap participates in conducting electricity. At the same time, the increase of electron energy will induce to change the conductivity of the dielectric material, namely, the radiation-induced conductivity [26]. The expression is as follows:

(1) $$\sigma_{r}=jm\mu \tau_{r}g_{f}=\frac{j\tau_{g}(D)GM\tau_{r}g_{f}}{100}\mu_{n}+\mu_{p}$$

in the formula: *j* is beam density; *g(D)* is the generation rate of electron-hole pairs; *G* is the yield of chemical radiation; *M* is the energy of the incident electron; *τ_r_* and *g_f_* indicate that the generated electron-hole pairs are recombined in the discharge probability *g_f_* after the average time *τ_r_*_;_ *μ_n_* is electron mobility; *μ_p_* is the hole mobility. With the increase of *M*, *g(D)* and *G* also increase. Therefore, *σ*_r_ and *g_f_* increase thereupon as well. On the other hand, the material undergoes micro-degradation after irradiation and the main chain of the organic polymer material breaks, resulting in side chains and side groups. At the same time, low molecular compound gas is produced. which escapes from the material surface. Micropore traps are formed on its surface, capture a large amount of space charge and make ionic conductance form. By this way, discharge power and discharge channel are provided, which accelerates the formation of discharge and causes multiple discharges.

Since the secondary electron emission coefficient of PI and other dielectric materials is less than 1 [27], when irradiated with high energy electron beam, electrons will gradually accumulate on their surfaces, causing the negative surface potential to increase gradually. At this time, an increasing electrostatic field is established between the dielectric surface and the background layer, whose direction is from the ground electrode to the surface of the medium. On the one hand, dark current will be generated inside the medium under the action of the electric field. When the dark current is less than the charging current of the electron beam on the medium surface, the surface charge of the medium will show a gradually upward trend [28]. On the other hand, due to the process problems in the production of dielectric materials, some clicks in the dielectric have lower penetration field strength than others, which may first establish discharge channels when the electrostatic field increases gradually. At the same time, the surface roughness of the dielectric is not uniform, resulting in higher surface potential in some parts of the dielectric material charged by the electron beam. The electrostatic field established under the action of its adjacent grounding electrodes is higher than that in other places, which may become discharge channels during the process of surface potential rising. When these discharge channels produce electrostatic discharges, the surface charge of the dielectric is discharged and the surface potential decreases. When the charge-discharge process occurs repeatedly and reaches the dynamic balance, the maximum surface potential of the medium also reaches a corresponding limit (surface charge balance potential).

Theoretically, due to the blocking effect of the dielectric surface potential on the electron beam current, the highest potential that can be reached on the dielectric surface is numerically equal to the energy of the electron beam when the electron beam charges the dielectric [29,30,31]. Under the electron beam irradiation with the same beam density, the increase of electron energy will lead to the decrease of radiation-induced conductivity [26]. Therefore, during the dynamic balance process of charge-discharge of dielectric materials irradiated by electron beam, with the increase of electron energy, the leakage current of dielectric decreases. When the equilibrium potential is reached, the amount of electronic charge accumulated on the dielectric increases, which leads to an increase in the surface balance potential. Previous experimental analysis shows that when there is external field radiation, induced electrostatic discharge will occur, which will increase the frequency of material discharge. At this time, during the charge-discharge balance process, the amount of charge accumulated on the media will decrease, resulting in the surface potential of the media decreased. Moreover, the stronger the external radiation field is, the faster the frequency of induced electrostatic discharge is. The faster the charge is discharged by electrostatic discharge, the less charge can be accumulated on the media surface, and the lower the media balance surface potential is.

#### 3.2.2. Comparative Analysis of Surface Charging Potentials of Different Thickness Dielectric under Intense Field

**Figure 9:**
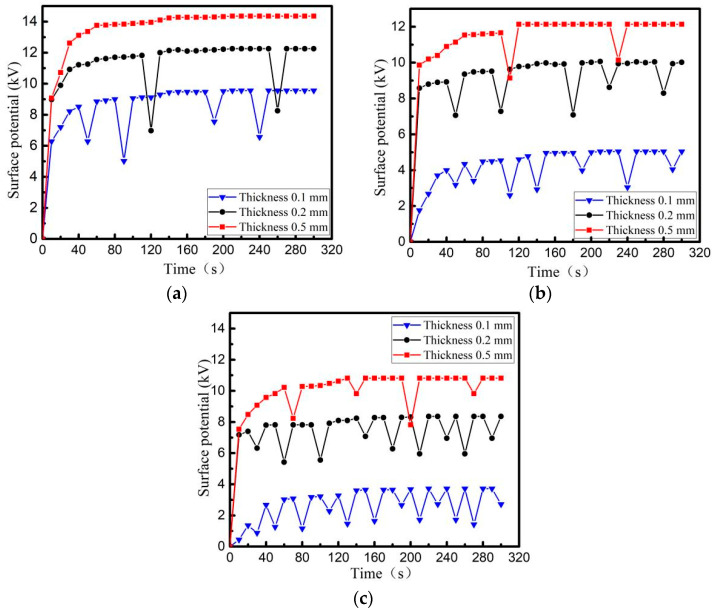
Surface potential of dielectric surface with different thickness without external field radiation (**a**) PTFE, (**b**) PI, (**c**) EP.

**Figure 10:**
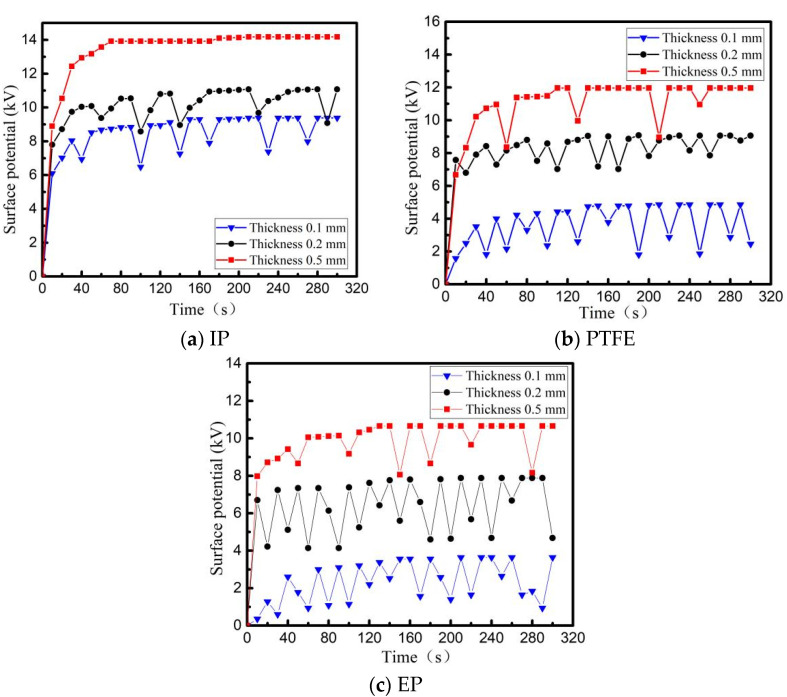
Surface potential of dielectric surface with different thickness irradiated by ESD field (**a**) PTFE, (**b**) PI, (**c**) EP.

Three dielectric materials: PI, PTFE and EP, whose thickness is 0.1 mm, 0.2 mm, 0.5 mm, are irradiated and charged, with a beam density of 5 nA/cm^2^ and an energy of 30 keV. The surface of the irradiated dielectric is 10 × 10 cm^2^ square, and back usage area is grounding Al-Pt with 5 × 5 cm^2^ square. The relationship between surface potential and time is shown in Figure 9. After the material surface is decomposed by plasma, ESD field with a peak field strength of 12.3 kV/m is used to irradiate the three media surfaces, and the relationship between the surface potential of the three media and time is re-measured as shown in Figure 10.

From Figure 9 and Figure 10, it can be seen that during the charging process using a high-energy electron beam, the final balance potential will increase with increasing dielectric thickness. Among the three materials with the same thickness, the PI charging potential is the highest and the number of discharges is the least under the same conditions. When using ESD field radiation media with peak field strength of 12.3 kV/m, the discharge number of other dielectric materials with different thickness, except for 0.5 mm PI, increases to different degrees, i.e., induced electrostatic discharges are produced by dielectric metal-coated electrodes under the action of radiation field. The thinner the material is, the more times electrostatic discharge will be induced.

Analysis: for the same kind of dielectric film with different thickness, the surface charge distribution is similar during the charging process of electron beam under the condition of the same surface roughness. However, due to the different thickness of the materials, the dielectric properties of them are different, that is, the thicker the material, the better the dielectric properties. As a result, during the dynamic balance process of charging-discharging dielectric materials with electron beam, the dielectric properties of the dielectric material increase with the increase of its thickness, and the leakage current inside decreases, resulting in a higher dynamic balance potential on the material surface.

#### 3.2.3. Comparative Analysis of Surface Charging Potentials of Different Dielectric Materials under Intense Field

Comparing the discharge characteristics of different materials in Figure 7, Figure 8, Figure 9 and Figure 10, it is concluded that EP has the highest discharge frequency and the lowest dynamic charging potential on its surface under the same conditions (whether or not there is an external radiation field). However, PI is just reverse.

Among the three materials, due to the smallest PI surface roughness and the largest EP surface roughness [32,33], the surface charge distribution of EP is the most uneven under the same charging conditions of electron beam irradiation. There may be the more discharge channels in the interior, the faster current release rate and the poorer ability of surface accumulative charge, thus the (dynamic) charging potential that EP material surface can reach is the lowest. Particularly, the above characteristics of PI material are exactly opposite to those of EP material. In addition, the dielectric constant can reflect the energy loss and storage capacity of the dielectric to the electrostatic field. The relative dielectric constant of PI, EP and PTFE is usually 4.8, from 3 to 4 and from 1.8 to 2, respectively [34]. For 0.5 mm PI thin film, its dielectric performance is better than the other two materials, so under the same charging condition, the leakage current inside is relatively small and the final surface balance potential is higher.

### 3.3. Study on Electrostatic Discharge Characteristics of Spacecraft Typical Media under Intense Field

#### 3.3.1. Effect of Radiation Field Strength on Electrostatic Discharge Frequency of Dielectric Material

**Figure 11:**
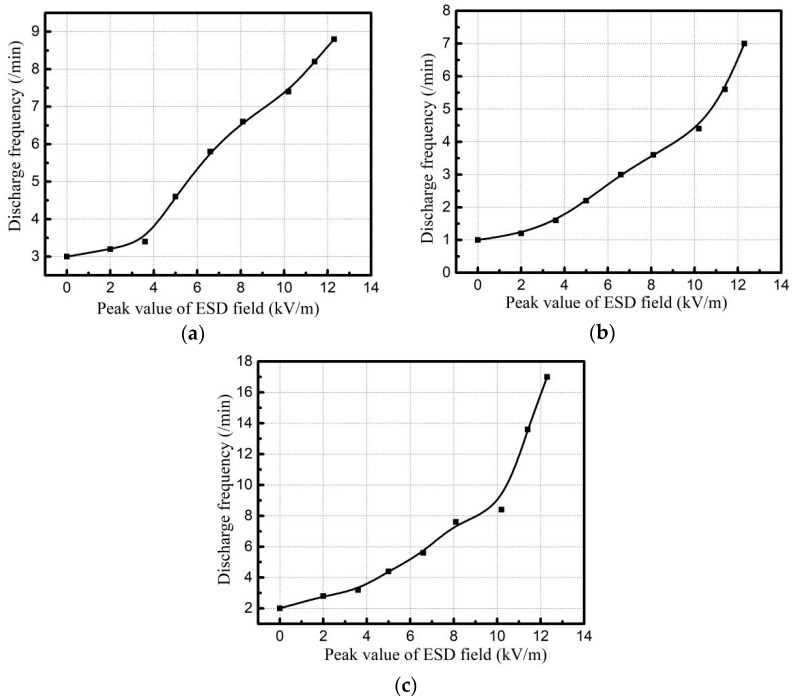
Variation of electrostatic discharge frequency peer minute with radiation field intensity of dielectric materials, (**a**) PI, (**b**) PTFE, (**c**) EP.

PI, PTFE and EP with 0.1 mm thickness are irradiated with an electron beam with energy of 30 keV and beam density of 5 nA/cm^2^. Al-Pt grounded on the back of specimens is 5 × 5 cm^2^ and the irradiated dielectric surface is 10 × 10 cm^2^. The relationship between the number of electrostatic discharges and the irradiation intensity of ESD field of three materials is shown in Figure 11.

From Figure 11, it can be seen that the discharge frequency of dielectric increases gradually with the increase of the irradiation intensity of ESD field during the electron beam irradiation. Comparing with the three media, it is found that under the same conditions, EP discharge is the most frequent in the three materials.

Analysis: in the process of electron beam irradiation on polymers such as PI, the electron-hole pair participates in the charge transport, resulting in radiation-induced conductivity [35]. When the electron energy is constant, increasing the electron beam density can improve PI-induced conductivity. When the beam current is constant, increasing the beam energy decreases the PI-induced conductivity [31]. Under the premise of unchanged intrinsic conductivity, the breakdown field strength of PI and other space media materials is in the order of 107 V/m [34], while the existence of electron-induced conductivity decreases the breakdown field strength. Similarly, when a dielectric material is irradiated by an external strong field, its internal structure polarizes, positive and negative charges are separated to form, and current is formed by carriers moving along the direction of electric field, which increases the conductivity of the material and further decreases the breakdown field strength of the dielectric. Therefore, when the external radiation field irradiates the back grounded media, the induced discharges will occur. The higher the external radiation field strength is, the more the evoked discharges per unit time.

#### 3.3.2. Effect of Dielectric Material Thickness on Electrostatic Discharge Frequency

**Figure 12:**
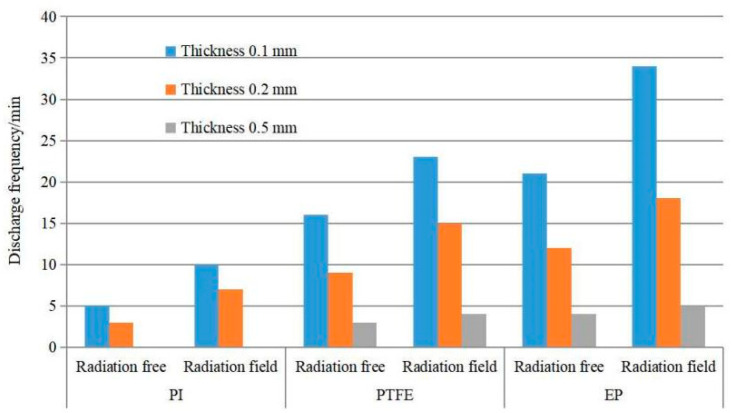
Comparison of the discharge of three materials with different thicknesses with or without external field irradiation.

PI, PTFE and EP with thickness of 0.1 mm, 0.2 mm, 0.5 mm are charged by an electron beam with beam density of 9 nA/cm^2^ and energy of 30 keV, respectively. The irradiated medium surface is 10 × 10 cm^2^ and the area of Al-Pt grounded on the back is 5 × 5 cm^2^, and the comparison of discharge frequency before and after ESD EMP irradiation is revealed in Figure 12.

From Figure 12, it can be seen that in the process of irradiating PI, PTFE and EP of different thickness with high energy of electron beam, all materials have different degrees of discharge except 0.5 mm PI, and the thinner the material, the more discharge frequency. When the charged dielectric material is irradiated by ESD field, its discharge frequency increases, that is, induced electrostatic discharge is produced by the action of radiation field. The thinner the thickness of the dielectric material, the more the number of evoked discharges. Comparing the discharging rules of different dielectric materials, it is found that EP has the highest frequency of evoked discharges and PI has the lowest one among the three materials under the same conditions.

Analysis: for the same kind of dielectric film with different thickness, the surface charge distribution is similar during the charging process with electron beam under the same surface roughness. However, the dielectric properties are different, which increases with the increase of the thickness of dielectric materials. The leakage current inside and the discharge frequency decrease, during the dynamic balance process of electron beam charging and discharging dielectric materials.

#### 3.3.3. Effect of Ground Electrode Shape on Electrostatic Discharge Frequency of Dielectric Material

**Figure 13:**
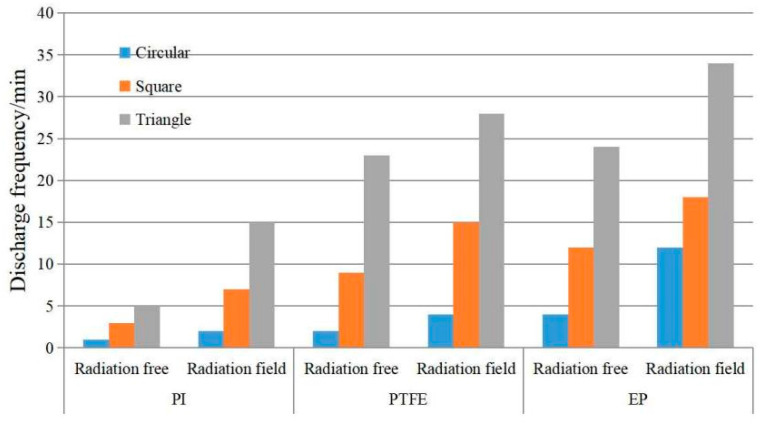
Comparison of discharge characteristics of dielectric materials coated ground electrodes of different shapes with or without external field irradiation.

PI, PTFE and EP are irradiated with a beam density of 9 nA/cm^2^ and an energy of 30 keV. The irradiation surface is 10 × 10 cm^2^ and 0.2 mm thick. The back ground Al-Pt has an area of 16 cm^2^, while the shapes are circular, square and triangular. The frequency comparison of the three dielectric thin films before and after ESD radiation field with a peak of 12.3 kV/m is shown in Figure 13.

From Figure 13, it can be seen that when PI, PTFE and EP are irradiated with high energy electron beam, the shape of the background Al-Pt affects the discharge frequency. The circle shape has the least discharge number, the square the second, and the triangle the most. When irradiated with an external radiation field, the discharge law of different shapes is similar to that without external radiation field, but the corresponding discharge number is obviously more.

Analysis: because the dielectric surface irradiated by the electron beam all is 10 × 10 cm^2^, it can be regarded as having the same charging rate. Due to the same grounding area of three shapes of grounding Al-Pt, the media area involved in discharge is basically the same. When the ground Al-Pt is triangle, its top angle shape is sharp. Under the action of dielectric surface charge, the local field strength distortion is more serious and the local field strength at the top angle is relatively high. When irradiated by electron beam, the induced conductivity of the media near the top angle is larger, the dielectric property of the material is decreased significantly, and the breakdown discharge is easier. Especially, induced discharges are more likely to occur. On the contrary, for a circular Al-Pt coated on the back of dielectric material, because there is no top angle, the field strength distortion at the Al-Pt edge is relatively weak, the conductivity of the material is relatively small, and the dielectric property of the material changes little. It is also relatively difficult to discharge under electron beam charging and ESD field irradiation. Moreover, the dielectric material with square-coated electrodes on the back has the intermediate difficulty degree of discharge among them.

#### 3.3.4. Effect of Ground Electrode Area on Electrostatic Discharge Frequency of Dielectric Material

**Figure 14:**
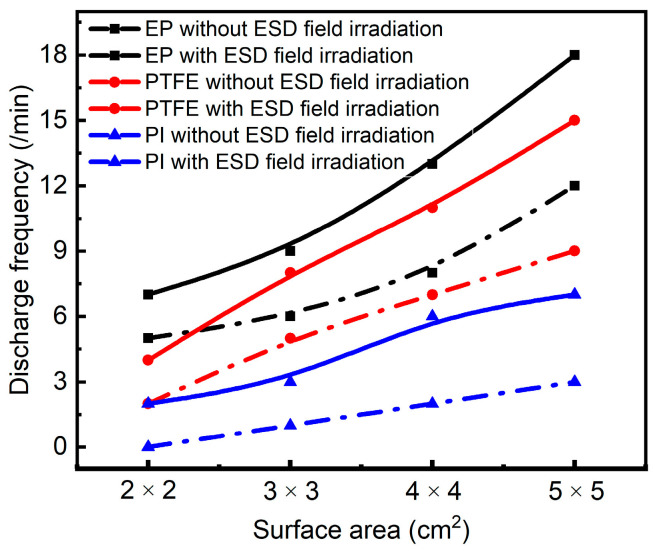
Discharge conditions of dielectric films with different grounding areas with or without external field irradiation.

PI, PTFE and EP, with the same thickness of 0.2 mm and different area of the background square Al-Pt electrode, are charged by electron beam with beam density of 9 nA/cm^2^ and energy of 30 keV. The irradiation surface is 10 × 10 cm^2^. The trend of discharge frequency of dielectric film of 12.3 kV/m along with the area of Al-Pt before and after ESD radiation with a peak value is shown in Figure 14.

It can be seen from Figure 14 that the ground area on the back affects the frequency of discharges. The smaller the background area, the less the number of discharges during the treatment of PI, PTFE and EP with high energy electron beams. When irradiated by radiation field, the larger the area, the easier the evoked discharge will be.

Analysis: For the same media, during the same electron beam charging process, it can be considered that the power-up condition is the same, that is, the surface potential of the media is the same. It can be further considered that the distribution of electrostatic field is similar when the background Al-Pt is of the same shape. However, the media area that can participate in discharge varies with the area of Al-Pt. Due to the defect of dielectric quality; there will inevitably be discharge channels with low breakdown field strength inside. The larger the media area, the more electrostatic discharge channels will be generated. Under the same electrostatic field, the more electrostatic discharges occur. Similarly, the number of evoked discharges increases with the irradiation of the radiation field.

## 4. Conclusions

Based on three typical dielectric materials commonly used in spacecraft, a research platform for charging and discharging experiments of dielectric materials in spacecraft under strong field was established. The influence factors of induced electrostatic discharge under strong field were studied, and the influence rules of irradiation field strength, beam energy, dielectric thickness, shape and area of ground electrode on charging and discharging of dielectric materials were summarized. It provides technical support for the static safety and protection of spacecraft.

1. Under vacuum conditions, when irradiated and charged with high-energy electron beam, the surface potential of PI, PTFE and EP increases gradually. Although the surface potential of the materials can decrease due to the charge discharge induced by electrostatic discharge, the surface potential of the media still increases gradually. Finally, a dynamic balance limit (surface charge balance potential) is reached. The surface balance potential increases with the energy of the irradiated electron beam.
2. Under vacuum conditions, when ESD irradiation is applied to charging material, the discharge frequency of material is increased, that is, the induced electrostatic discharge is produced by the dielectric material under the action of the radiation field. With the increase of radiation field strength, the number of evoked electrostatic discharge increases, the accumulated charge on the dielectric surface decreases, and the dielectric surface potential decreases.
3. Under vacuum conditions, under the same charging mode and radiation conditions, the discharge frequency of PI is the lowest, PTFE next, EP the highest, inversely the surface dynamic balance potential of EP is the lowest, PTFE centred, PI the highest. The same change of the three media is that the thicker the dielectric material, the lower the discharge frequency and the higher the surface dynamic balance potential.
4. Under vacuum conditions, when high-energy electron beam charges a dielectric material, the shape and size of the background electrodes will affect the discharge frequency of the dielectric material. When the area of the electrodes is the same, the sharper the electrode edge, the higher the discharge frequency. When the electrodes are of the same shape, the larger the grounding area, the higher the discharge frequency.

## Figures and Tables

**Table 1:** Peak value of ESD field produced by different discharge gun voltages.

Discharge Gun Voltage (kV)	−1	−5	−10	−15	−20	−25	−30
Radiation peak field intensity (kV/m)	−0.5	−3.6	−6.6	−8.1	−10.2	−11.4	−12.3

## Data Availability

The data presented in this study are available on request from the corresponding author.
